# Modeling the Contributions of Basal Ganglia and Hippocampus to Spatial Navigation Using Reinforcement Learning

**DOI:** 10.1371/journal.pone.0047467

**Published:** 2012-10-26

**Authors:** Deepika Sukumar, Maithreye Rengaswamy, V. Srinivasa Chakravarthy

**Affiliations:** 1 Cognizant Technology Solutions, Bangalore, India; 2 Department of Biotechnology, Indian Institute of Technology Madras, Chennai, India; University of Chicago, United States of America

## Abstract

A computational neural model that describes the competing roles of Basal Ganglia and Hippocampus in spatial navigation is presented. Model performance is evaluated on a simulated Morris water maze explored by a model rat. Cue-based and place-based navigational strategies, thought to be subserved by the Basal ganglia and Hippocampus respectively, are described. In cue-based navigation, the model rat learns to directly head towards a visible target, while in place-based navigation the target position is represented in terms of spatial context provided by an array of poles placed around the pool. Learning is formulated within the framework of Reinforcement Learning, with the nigrostriatal dopamine signal playing the role of Temporal Difference Error. Navigation inherently involves two apparently contradictory movements: goal oriented movements vs. random, wandering movements. The model hypothesizes that while the goal-directedness is determined by the gradient in Value function, randomness is driven by the complex activity of the SubThalamic Nucleus (STN)-Globus Pallidus externa (GPe) system. Each navigational system is associated with a Critic, prescribing actions that maximize value gradients for the corresponding system. In the integrated system, that incorporates both cue-based and place-based forms of navigation, navigation at a given position is determined by the system whose value function is greater at that position. The proposed model describes the experimental results of [1], a lesion-study that investigates the competition between cue-based and place-based navigational systems. The present study also examines impaired navigational performance under Parkinsonian-like conditions. The integrated navigational system, operated under dopamine-deficient conditions, exhibits increased escape latency as was observed in experimental literature describing MPTP model rats navigating a water maze.

## Introduction

Animal navigation is assisted by a combination of wandering exploratory movements and goal-directed movements. Animals tend to adopt one of three different navigation strategies – 1) taxon navigation, 2) praxic navigation and 3) locale navigation – according to the environment, the task at hand and the inputs that they receive from the environment [2]. In *taxon* navigation the animal simply pursues a visible goal represented in an egocentric coordinate system. In *praxic* navigation, the animal codes the trajectory in terms of body turns triggered by external stimuli (e.g, turn right at the next junction). In *locale* navigation, the animal uses, not just a single cue but a much larger spatial context, to construct its own internal map of surrounding space, and navigates thereby. Taxon navigational strategy is also called cue-based navigation and locale-based navigation is also known as place-based navigation [3], which is the terminology used in this paper. More recent models have shown that spatial navigation with a topological map is more suitable to explain highly flexible navigational behavior, a capability that is not afforded by purely distance-based models [4]–[7].

Experimental work on spatial navigation in animals suggests that, the basal ganglia are recruited for the encoding of stimulus-response or cue-based form of navigation [8]. Hippocampus provides spatial information, generating a cognitive map of the environment, thereby subserving place-based navigation [9]. The two neural substrates receive different inputs, process different representations and operate in parallel to support navigation [10]. Functional neuroimaging studies on human beings have revealed that the relative contribution by each system depends on the strategy chosen by the agent [11]. Experiments suggest that in rodents, these two systems interact competitively during navigation [12]. Also, there is evidence for the two systems being employed successively at different stages of proficiency [13]. Hence, a realistic model of animal navigation should demonstrate both the strategies and incorporate an appropriate switching mechanism between the two.

Early lesion studies on the distinctive roles of Basal ganglia and Hippocampus in spatial navigation may appear to give a simplified picture of the matter: place-based navigation of Hippocampus in the early stages, followed by cue-type navigation of Basal ganglia [14]. However, Basal ganglia and Hippocampus are not simple, unitary entities but complex circuits involving several nuclei or subregions. The Basal ganglia are a group of subcortical nuclei comprising the striatum (caudate-putamen), the external and internal segments of the globuspallidus (GPe and GPi), the subthalamic nucleus (STN) and the substantia nigra (SN) [15]. The SN is further split into two nuclei: the pars compacta (SNc) containing dopaminergic neurons and the pars reticulata (SNr), an output nucleus of Basal ganglia. Similarly, the hippocampal formation comprises the entorhinal cortex, dentate gyrus, fields CA3 and CA1 of the hippocampus proper and the subiculum. These form a loop of connections that starts and ends in the entorhinal cortex [16].

Spatial navigation of various forms seems to be a result of complex interactions among different components of basal ganglia and Hippocampus. For example, dorsal medial striatum in Basal ganglia is thought to receive place-coding information from hippocampus [1], [8]. Similarly lateral dorsal striatum is thought to have a role in selecting stereotyped responses in cue-based navigation [1], [8]. Thus an ideal computational model of spatial navigation should be able to explain the relative contributions of various Basal ganglia and hippocampal regions to spatial navigation.

There are models of navigation that incorporate both Basal ganglia and Hippocampus, and accommodate both cue-based and place-based navigational forms respectively [5], [17], [18]. However; existing models do not seem to identify the neural substrate for exploratory dynamics necessary for navigation. But this is important since navigation is a combination of goal-directed movements and wandering, exploratory movements. It has been proposed that the indirect pathway of Basal ganglia is the substrate for exploratory drive [19]–[23]. With this assumption about the role of the indirect pathway, it seems to be possible to explain the manifold functions of Basal ganglia (action selection, working memory, motor preparation, goal-oriented behavior, sequence generation etc.) in a single modeling framework [19]. Most existing models of Basal ganglia tend to focus on one or two functions of basal ganglia, while ignoring others. Our effort is to show that the same model can be used to explain the whole range of models, which is a much harder accomplishment than concocting a different model for every function. The present model is one such a development of our core Basal ganglia model, which aims to describe the contributions of basal ganglia, along with hippocampus, to spatial navigation.

In the present work, we describe an integrated model of spatial navigation involving both Basal ganglia and hippocampus and hypothesize that the indirect pathway of Basal ganglia provides the exploratory drive for navigation. The proposed model integrates the above two forms of navigation – cue-based and place-based – into a single framework. It also incorporates mechanisms of gating between the two forms of navigation, as it happens perhaps in a real navigating rat. Furthermore, with an explicit representation of dopamine signal, the model provides an opportunity to study navigation under dopamine-deficient conditions as in the case of Parkinson's disease.

The model is cast in the framework of Reinforcement Learning (RL), a branch of machine learning [24]. Model performance is tested on a simple simulated Morris water maze, in which a model rat searches for a platform, visible or hidden. Contributions from Basal ganglia to this process consist in three things: 1) in modeling the value, the reward-yielding potential, of the current position of the rat, 2) in supplying the stochastic perturbations necessary to drive the wandering, searching movements of navigation, and 3) in using the reward information received, whenever the model reaches the platform, to drive navigation. Like in several other RL-based models of basal ganglia [25], [26] the temporal difference error (TD error), which is defined as the difference between the predicted total future reward and the actual future reward, represents the activity of mesencephalic dopaminergic neurons. Contributions from hippocampus, in this model, consist of representing the surrounding space as a topographic map of views. Contributions from basal ganglia and hippocampus to navigation problem are incorporated in a single model. Basal ganglia and hippocampus components compete to drive navigation in the integrated model.

The paper is organized as follows. Sections 2 describes the integrated model which combines the cue-based and place-based modules. Section 3 presents the simulation results applied to two experimental conditions: 1) the navigation study of [1], and 2) navigation under Parkinsonian conditions. A discussion of the model results along with possible future directions is presented in the final section.

## An Integrated Model For Navigation

In circumstances wherein both cue-based and place-based strategies coexist, the two strategies may compete with each other during learning [27], [28]. Evidence shows that there is interference from hippocampus-dependent learning in basal ganglia-dependent processes. In rats, lesions in hippocampus enhance acquisition of Basal ganglia-dependent navigation strategy in a radial arm maze task [29], [30]. Hence, the two systems may be recruited in parallel, with different parts of basal ganglia or hippocampus participating in different kinds of navigation.

**Figure 1:**
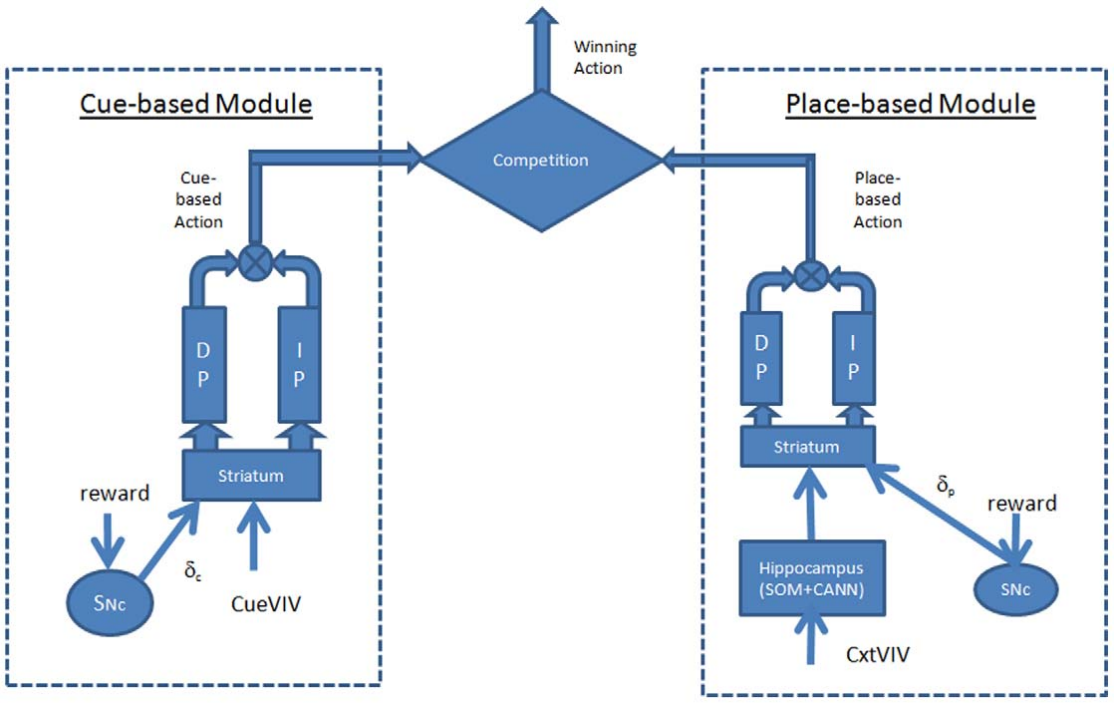
Architecture of the integrated model that combines both navigation strategies. It combines a Cue-based and a Place-based navigation module. Each module consists of a unique Critic and a Temporal Difference error signal. Whereas the Cue-based module depends on the visible target, the Place-based module depends on the spatial context. One of the modules is selected at any given instant, by comparing the values estimated by the two modules.

A complete model of animal navigation must include both cue-based and place-based responses. A competition mechanism must be set up to select the action to be performed. Hence, the integrated model developed includes a cue-based and a place-based module (fig. 1), competing with each other to assist navigation. The architectures of the cue-based and place-based modules are now described.

### 2.1. The Cue-Based Module

In cue-based navigation the animal directly homes in on a visible target. Spatial representations in hippocampus are not thought to be involved in this type of navigation, which is also called Stimulus-Response (S-R) type of navigation since it involves response by movement to the stimulus of a visible target. This S-R type of navigation strategy is thought to be subserved by the basal ganglia region of the brain [31], [32].

Basal ganglia have been known to be associated with control and selection of voluntary behavioral acts [33]. They are actively involved in resolving conflicts between multiple sensorimotor systems seeking access to a common motor path [34]. Hence they enable animals to select appropriate actions under dynamic sensory and motivational conditions. Experiments suggest the involvement of cortico-basal ganglia-thalamocortical circuit in preparation of externally cued movements [35].

**Figure 2:**
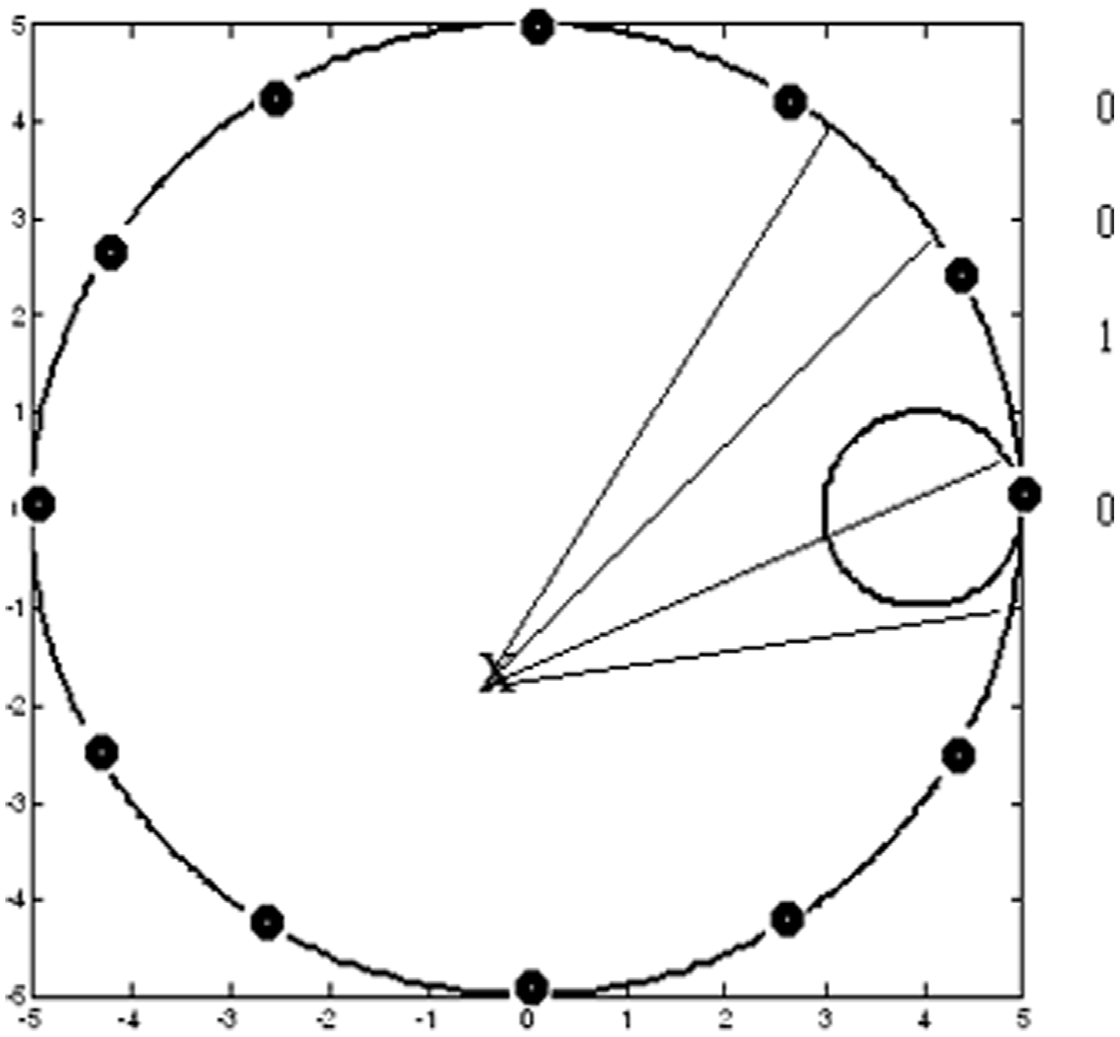
View-vector construction in case of cue-based navigation. The platform is the smaller circle to the right of the circular pool. (The black dots on the rim are poles used for place-based navigation. Not all poles are shown.) The field of view (angle of vision = 180°) is divided into 30 sectors. Sectors that intersect with the platform are associated with a value of 1; those that do not intersect are given a value of 0. Thus a 30-dimensional binary Cue-based Visual Input Vector is constructed.

This model of navigation is instantiated in a simulated rat exploring a simulated circular Morris water maze (radius = 5) (fig. 2). There is a circular platform of radius 1, towards the right of the pool. The simulated rat is trained to directly identify and approach the visible platform – the cue.

The appearance of the visible platform, which is coded in the form of Cue-based Visual Input Vector (fig. 2), is given as input to basal ganglia. The basal ganglia system is responsible for computing the value function and for producing the exploratory drive. During the course of exploration, whenever the model rat arrives at the pool, accidentally, it receives a positive reward (+1), which is used to train the value function. If the model rat bumps into the wall of the pool, it is given a negative reward (−1) and its position and orientation are randomly re-initialized. Elsewhere in the pool, reward is zero. During training, if the model rat cannot find the platform within 400 steps the trial is aborted and the model rat is re-initialized to a random position and orientation. We will show how the machinery of Basal ganglia can be used to perform hill-climbing over the trained value function, thereby enabling the model rat to move towards the platform.

In order to construct the Cue-based Visual Input Vector, the field of view is divided into 30 sectors. Sectors that intersect with the platform are associated with a value of 1; those that do not intersect are given a value of 0 (fig. 2). Thus a 30-dimensional binary Cue-based Visual Input Vector is constructed.

#### Model of Basal Ganglia

**Figure 3:**
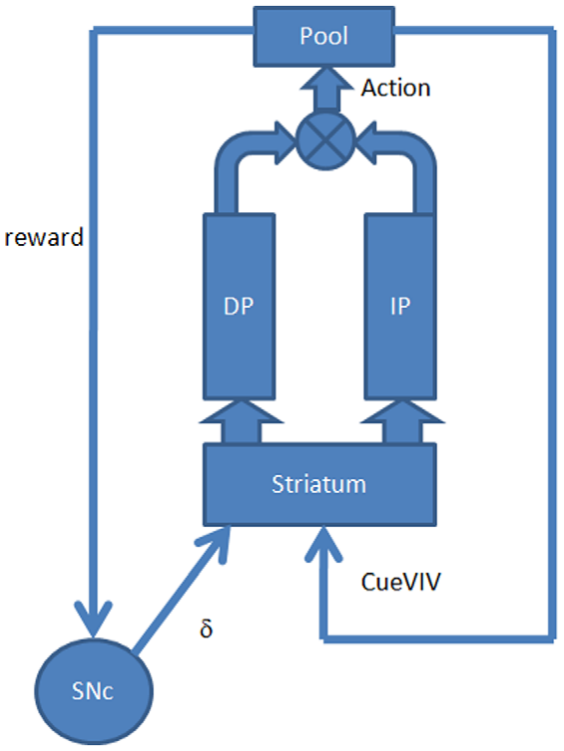
Architecture of the module for cue-based navigation. The striatum receives dopamine signal from SNc, and the sensory input Cue-based Visual Input Vector, which is processed by Direct Pathway and Indirect Pathway. Value is computed in striatum. Outputs of Direct Pathway and Indirect Pathway are combined to compute the action, which represents the displacement (Δz = [Δx, Δy]) of the simulated rat in the next step.

In case of cue-based navigation, movements of the rat are controlled by the output of Basal ganglia, which consists of the combined outputs of the Direct Pathway and the Indirect Pathway (fig. 3). Direct Pathway and Indirect Pathway responses are modeled as a function of both, phasic (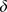) and tonic dopamine (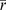), as described below.

#### Dopamine signal

Dopamine fluctuations are often classified as *phasic*, which refers to changes over seconds, or *tonic*, which refers to fluctuations over a time-scale of minutes [36].

#### Phasic dopamine

The work of [37], suggests that phasic dopamine signals arising from neurons of mesencephalic brain regions may be interpreted as the temporal difference (TD) error, which stands for the difference between the total actual future reward and the total predicted future reward. This signal, which is used for training the value function, is calculated as:
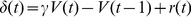
(2.1)where

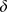
 is the temporal difference in Value,

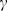
 is a constant discounting factor,

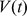
is the Value at time t,

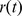
is the reward received at time t.

#### Tonic Dopamine

Tonic dopamine denotes a long-term baseline firing rate of mesencephalic dopamine cells. Tonic dopamine level is represented in the model by a discounted integration of phasic dopamine signal (eqn. 2.2):
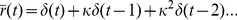
(2.2)where

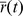
 is the tonic dopamine level at time 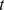,

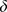
's are the TD errors (phasic dopamine), 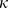 is a constant factor (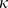
<1)

The values of various parameters used in this model are given in Appendix S1.

#### Direct and Indirect pathways (Direct Pathway and Indirect Pathway)

Dopamine signal or the TD error signal modulates activity in Basal ganglia, and controls its output. Classically, since activation of Direct Pathway was thought to result in facilitation of cortically initiated movements, Direct Pathway was termed the “Go” pathway, while the Indirect Pathway was termed the “NoGo” pathway since its activation inhibits movement [38]. Striatal dopamine is thought to switch between Direct Pathway and Indirect Pathway and therefore, between Go and NoGo regimes. Recently we had proposed that the classical Go/NoGo dichotomy may have to be expanded to Go/Explore/NoGo, with the Explore regime subserved by the complex dynamics of the Indirect Pathway neurons [19], [21], [22], In the new system, Direct Pathway and Indirect Pathway are selected for high and low dopamine levels respectively; but for moderate dopamine levels exploration takes place subserved by Indirect Pathway. (In RL literature, when the agent chooses an action that has the highest Value, it is said to be ‘exploiting’; if it is trying out actions that are known to be suboptimal, it is ‘exploring’.) In the present model, since the action space is continuous, exploitation and exploration are combined with a weighting factor that is dependent on dopamine signal.

The Direct Pathway and Indirect Pathway responses are modeled as a function of both phasic dopamine, (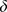) and tonic dopamine (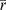).
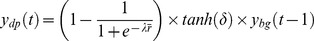
(2.3)

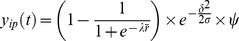
(2.4)

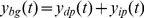
(2.5)

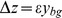
(2.6)where,

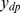
 is the output of the Direct Pathway,

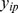
 the output of the Indirect Pathway,

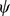
 a noise term of variance ^1^ and mean 0, arising out of Indirect Pathway,

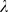
 a constant that controls the slope of the sigmoid function,

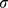
 the standard deviation of the Gaussian.

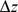
 the update for the position of the model rat

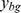
 total output of Basal ganglia

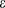
- a constant that converts Basal ganglia output to position update

Let us consider a verbal description of the dynamics of eqns. (2.3–2.6). When 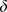
is positive and high (i.e. current position is better than the previous one with respect to the goal), it indicates that the rat is proceeding towards the goal. Hence, exploitative behavior (following the Value gradient) is required and not exploration. In this scenario, Direct Pathway response is high, and the model rat continues in the same direction as in the last step. However, if 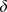
is negative and high, it indicates that the rat is proceeding in the direction that is perhaps opposite to the direction which would lead to the goal. The direction of navigation of the rat should now be reverted. To account for these two conditions in the model, Direct Pathway response is modeled as a sigmoidal function of 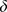
(eqn. 2.3). If 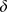 is low in magnitude, it indicates that the rat could not make much progress towards (or away from) the goal in the previous step. In this situation, more of exploratory behavior is required. The rat should be free to explore new directions. Hence, Indirect Pathway response is modeled as Gaussian noise, that is a function of 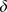, with 0 mean (eqn. 2.4).When 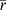 value is high, it is likely that the rat has almost reached the goal and is probably satiated. Therefore, both Direct Pathway and Indirect Pathway outputs must be low. Movements of the simulated rat should be dominated by combined Basal ganglia output. To account for this in the model, Indirect Pathway and Direct Pathway responses are modeled as sigmoidally decreasing functions of 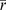 (see eqns. (2.3,2.4)).The final Basal ganglia output is the sum of the respective outputs of Direct Pathway and Indirect Pathway (eqn. 2.5). The update to the position of the model rat is simply a scaled version of Basal ganglia output (eqn. 2.6).

#### Training

Training of the critic network in this model, which is represented by a two-layer perceptron, is described by the following equations:
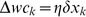
(2.7)

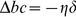
(2.8)where,

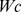
 - weights between the input layer and the single output node in the Critic network,
b_c_ – bias at the output node
δ – TD error as defined in eqn. (2.1)

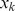
 - k'^th^ component of the feature vector Cue-based Visual Input Vector

### 2.2. The Place-Based Module

In place-based navigation the animal navigates with the help of an internal spatial model, which is constructed out of the spatial context of the world. Information regarding spatial context is combined with information derived from path-integration. However, path-integration is not incorporated in the present model. This internal model of surrounding space is thought to be represented in the hippocampus. The model presented in this section describes how basal ganglia and hippocampus work together in place-based spatial navigation.

**Figure 4:**
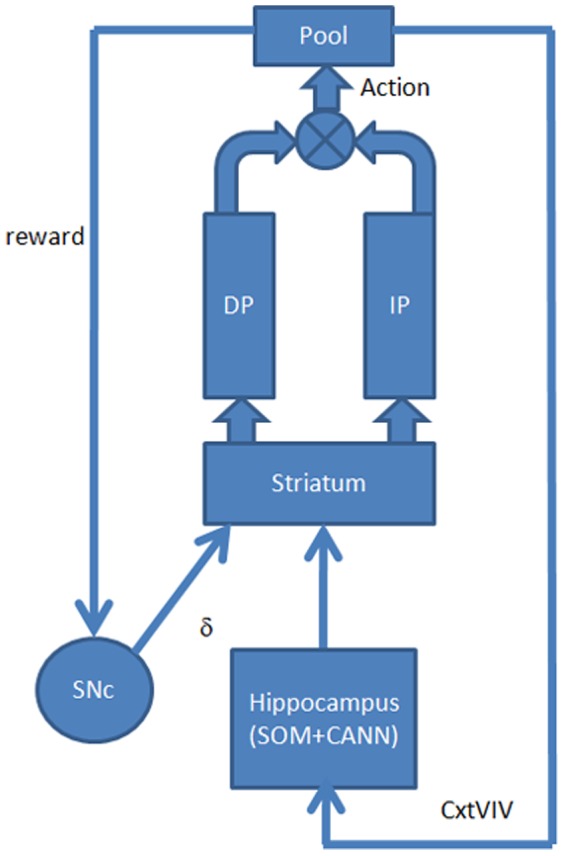
Architecture of the place-based module. The striatum receives dopamine signal from SNc, and the sensory state from Hippocampus, which is processed by Direct Pathway and Indirect Pathway. Value is computed in striatum. Outputs of Direct Pathway and Indirect Pathway are combined to compute the action, which represents the displacement (Δz = [Δx, Δy]) of the simulated rat.

**Figure 5:**
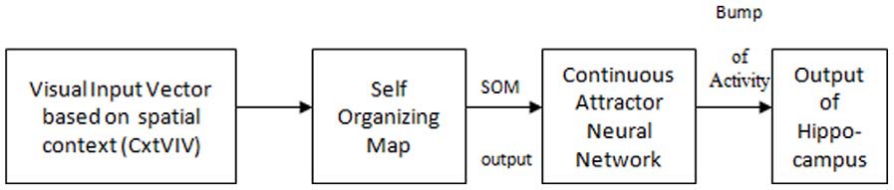
Representation of the modules that constitute the hippocampus. Visual input from the spatial context is presented to the SOM. Output of the SOM is the input to the CANN. CANN output is presented as input to the Basal ganglia in the place-based module.

The model architecture for place-based or context-based navigation (fig. 4) is similar to the one described in Section 2.1 (fig. 3). An additional element included in this model architecture is the hippocampus represented by a combination of Self Organizing Map (SOM) [39], and a Continuous Attractor Neural Network (CANN) [40] (fig. 5).

In the present model, the simulated rat has to explore a pool of muddy water, searching for a submerged platform. The simulated rat navigates with the help of spatial context, which is provided by an array of poles of equal height, placed around the pool (fig. 2).

The visual input presented to the rat is a set of heights of retinal images of landmark (a set of 24 poles, each of height 6, placed around the pool), which are viewed by the simulated rat from a certain vantage point. The height of the retinal image of each pole, which lies within the visual field of the simulated rat, is calculated according to eqn. (2.9).
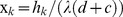
(2.9)where,

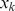
 is the height of the retinal image of k'th pole, viewed by the model rat

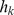
 is the actual height of the k'th pole ( = 6 for all k),

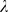
 is a constant (0.01),

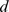
 is the distance between the simulated rat and the pole

c – a constant added in the denominator of RHS, to ensure that x_k_ does not blow up in close proximity to a pole. (c = 1)

The array of values x_k_ constitute the Context-based Visual Input Vector.

**Figure 6:**
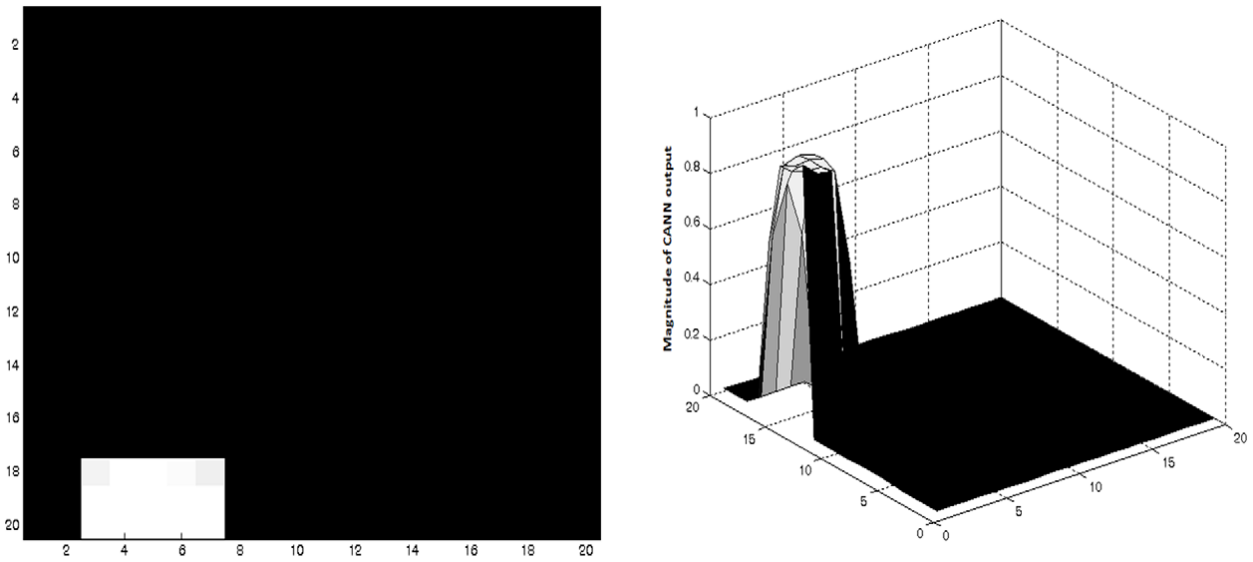
(a) A snapshot of the SOM response for a given Context-based Visual Input Vector. (b) The corresponding CANN response for the same Context-based Visual Input Vector.

A set of these Context-based Visual Input Vectors constructed for a set of random positions and orientations are used to train a two-dimensional self-organizing map [39], of size 20×20. For each visual Context-based Visual Input Vector presented, the output from the SOM is given as the input to a continuous attractor neural network (CANN) [40], also of size 20×20. Figs. 6 a,b show the corresponding responses of SOM and CANN to a given Context-based Visual Input Vector. Justification for use of a combination of SOM and CANN for modeling Hippocampus is presented in the discussion.

The output of the CANN is presented as input to the basal ganglia module of the previous section. Thus Basal ganglia drives navigation based on the place-based information obtained from Hippocampus. As in the cue-based model, the Direct Pathway and Indirect Pathway responses are modeled as a function of both, phasic dopamine, 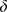, and tonic dopamine, 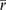. Direct Pathway response is modeled as a sigmoidal function of 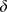 (eqn. 2.3). Indirect Pathway response is modeled as a Gaussian function of 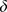, with the mean at 0 (eqn. 2.4). Indirect Pathway and Direct Pathway responses are modeled as sigmoidally decreasing functions of 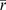 (eqns. 2.3 and 2.4). The next step of the model rat is a scaled version of Basal ganglia output (eqn. 2.6). As in the cue-based case, the place-based critic also is modeled as a perceptron and trained similar to the way the critic in cue-based case was trained (eqns. 2.7,2.8).

### 2.3 Architecture of the Integrated Model

The integrated model developed includes two critic networks, one for the cue-based critic and the other for the place-based modules (fig. 1). Separate visual input vectors are presented to the two modules: visual input representing the context information Context-based Visual Input Vector to the place-based module and the visual input regarding the visible platform Cue-based Visual Input Vector to the cue-based module. The Basal ganglia is also modeled separately for the two modules and its output is calculated from both modules. At each position of the model rat, the values from the two critics corresponding to the two strategies are compared and weighted by a selection parameter (g).The parameter g is a slowly changing function of the past navigational choices made by the animal such that the previously selected mode of navigation is more likely to be selected again (similar to gating mechanism used in [17]. It is incremented as follows:
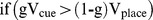
then
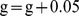
else
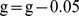



The next step of the simulated rat is determined by a softmax selection applied to the weighted values of the two strategies (V_cue_ and V_place_) as follows [41]:
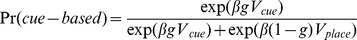
(2.10)where Pr(cue-based) is the probability of selecting the cue-based strategy, and ‘β’ is the exploration parameter.

## Comparison With Experimental Results

The integrated model of Section 2 is applied to explain two experimental conditions. These results are now described.

### 3.1. Simulating the Experimental Study of [10]

#### Case 1: Both strategies are used in competition with each other

An experimental training and evaluation procedure for simultaneous learning by both cue- and place-based modules was devised by [1]. In this experiment, rats were trained for 9 days, with interleaving trials involving the visible and hidden versions of the water maze. On days 1, 2, 4, 5, 7 and 8, the rat was trained to navigate to a visible target. On days 3, 6 and 9 of the training phase, the platform is made invisible; the rat then had to navigate with contextual spatial cues. The location of the platform, visible or invisible, is held invariant throughout the training.

**Figure 7:**
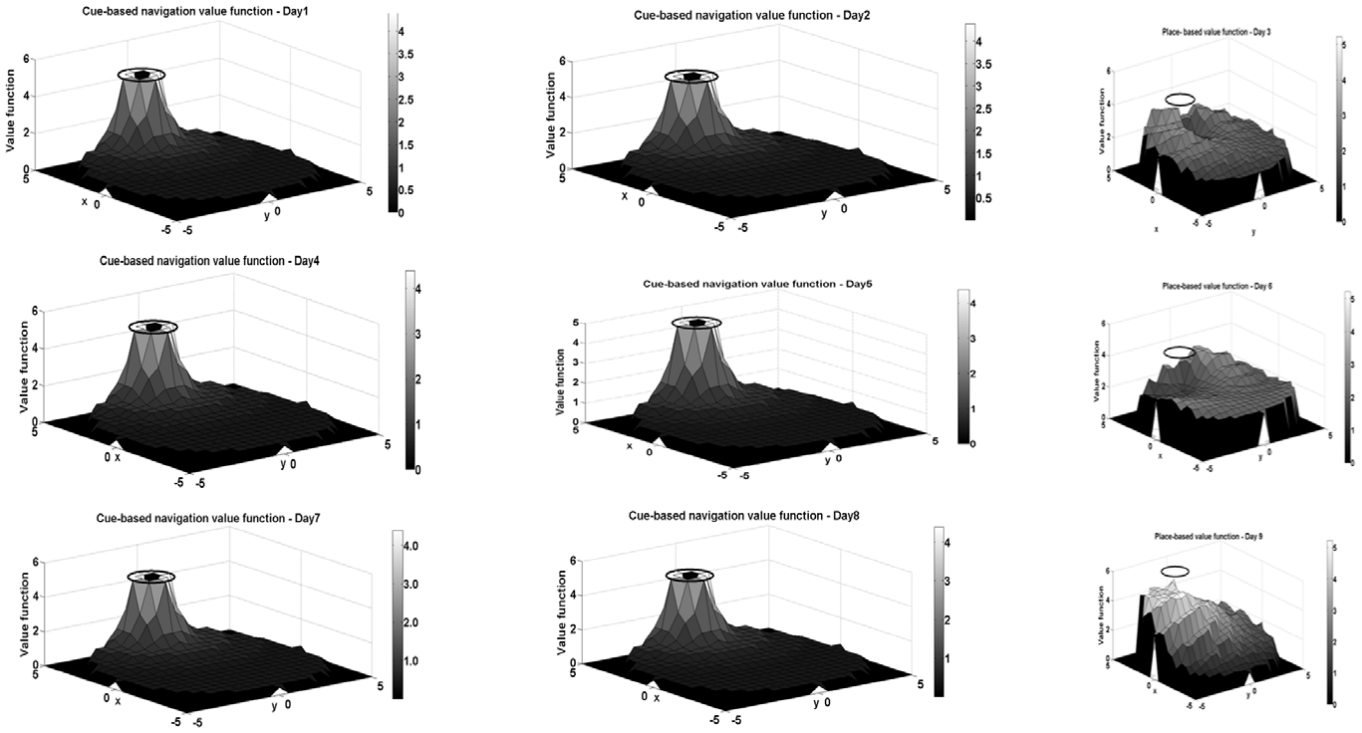
Critic profiles obtained on (a)cue-based module on Day 1, (b) cue-based module on Day 2, (c) place-based module on Day 3, (d) cue-based module on Day 4, (e) cue-based module on Day 5, (f) place-based module on Day 6, (g) cue-based module on Day 7, (h) cue-based module on Day 8, and (i) place-based module on Day 9. Note that both place-based and cue-based modules are trained on days 1,2,4,5,7,8, though only value profiles of cue-based module alone are shown. The value for both modules is a function of a high-dimensional vector. For ease of presentation, the value show in the above plot corresponds to a given position of the simulated rat, when the rat is oriented towards the center of the platform.

Along similar lines, an interleaving training procedure is applied to the model also. The platform is maintained at the same location, to the right of the pool, as in fig. 2. Each ‘day’ corresponds to 40trials of training in the model. Training on various days is conducted as follows. On days 1,2,4,5,7 and 8, the integrated model is used to train the animal using 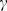 = 0.95. Fig. 7 a,b,d,e, g and h show the value profile of the cue-based module at the end of each of these days. As can be seen from fig. 7(a), the value function of the cue-based module is already well trained at the end of day 1. Since the animal was trained with an invisible platform on days 3, 6 and 9 in the corresponding model situation, the critic network of the place-based module alone is trained on these days using 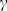 = 0.95. The corresponding value function profiles of the place-based module are shown in fig. 7c, f and i. On the other days, both value functions are trained and allowed to compete with each other. The trained weights of the place-based critic of one day are carried forward to the next day for further training. In case of the weights of the cue-based critic also, the same procedure is followed except on day 4, 7 and 10 when the weights of the critic from days 2, 5 and 8 are used respectively (since only place based critic is trained on days 3, 6 and 9). On day 10, the platform is shifted to the left and allowed to be visible. For this day, two cases were considered – one where the integrated model is used and the other where only the cue-based critic is used. In both these cases, no further training of weights was carried out. The values of various parameters used in this model are given in Appendix S1.

**Figure 8:**
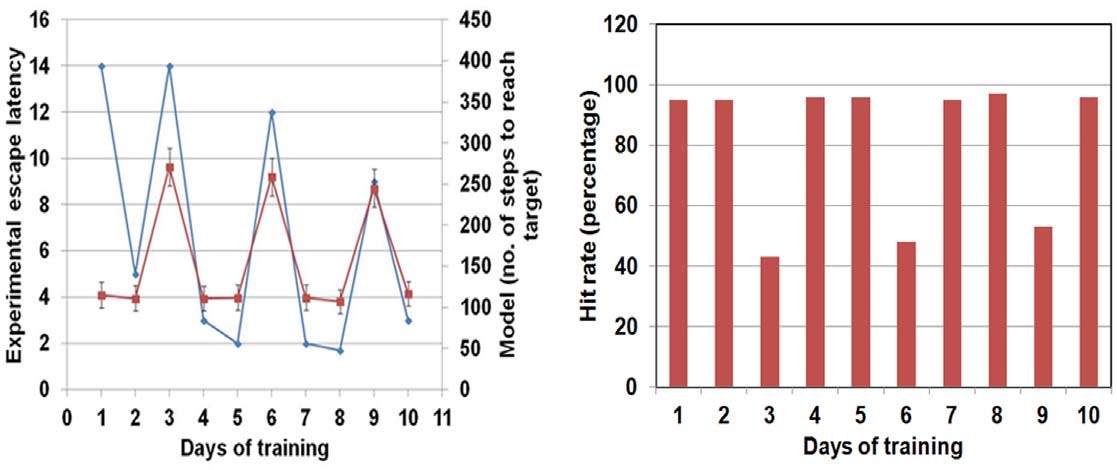
(a) Comparison of the escape latency of the agent in the experimental set-up, shown in seconds [1], and that of the simulated rat on different days of training shown as number of steps to reach the platform. (b) The hit rate of the model rat (expressed as percentage) on the different days of training.

Performance of the simulated rat is compared with the experimental results of [1], in fig. 8.Performance of the animal in experiments is quantified in terms of escape latency (fig. 8a), which denotes, in the model, average number of steps taken by the agent to reach the platform (shown on the secondary y-axis in red) and compared with the experimental escape latency measured in seconds (shown on the primary y-axis in blue). As can be seen from fig. 8a, the model and the experimental results both show very similar trends qualitatively. The main difference being that the model learns very fast on the first day itself and reaches minimum escape latency. A similar trend can also be seen from the plot of hit rate on the different days of the trial (fig. 8 b). Hit rate represents the percentage of successful trials in a block where the model rat reaches the platform. As can be seen from this plot, the model rat learns to find the platform very successfully on day 1 itself, and does not show much change in its success on later days. On days 3, 6 and 9, when the platform is not visible and only place-based module is used for navigation, a steady improvement is seen in the number of successful trials indicating that the place-based critic is learning though at a slower rate than the cue based one.

**Figure 9:**
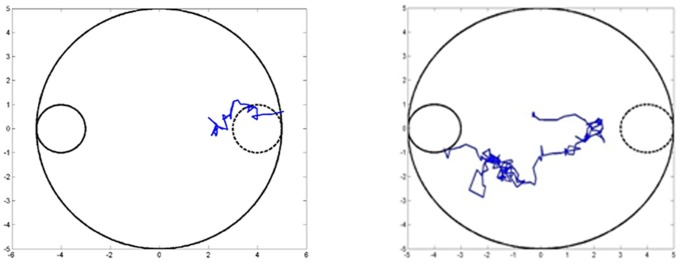
(a) Sample trajectory of the model rat going to the old location of the platform due to dominance of place-based trajectory on the 10^th^ day. (b) A sample trajectory in which the model rat first goes towards the old platform and then goes to the new platform location on the 10^th^ day.

On the tenth day, again, both place and cue-based strategies are used in competition with each other but the location of the platform is changed and the platform is now visible. However, this does not decrease the success rate of the model animal or increase the number of steps taken to reach the platform very much. It should be noted that no further training of either the cue-based or the place-based critic is carried out on this day and the results are due to the already trained weights. A further analysis of the model shows a clear domination of the cue-based navigation strategy over the place based with the cue-based being used as the major strategy in 35 trials out of 50. Fig. 9 shows two sample trajectories of simulated rat: in fig. 9a the simulated rat goes to the old location due to predominance of place-based navigation, and in fig. 9b the model rat first tries to go the former location and then approaches the current location.

#### Case 2: Only cue-based strategy is used to assist navigation

**Figure 10:**
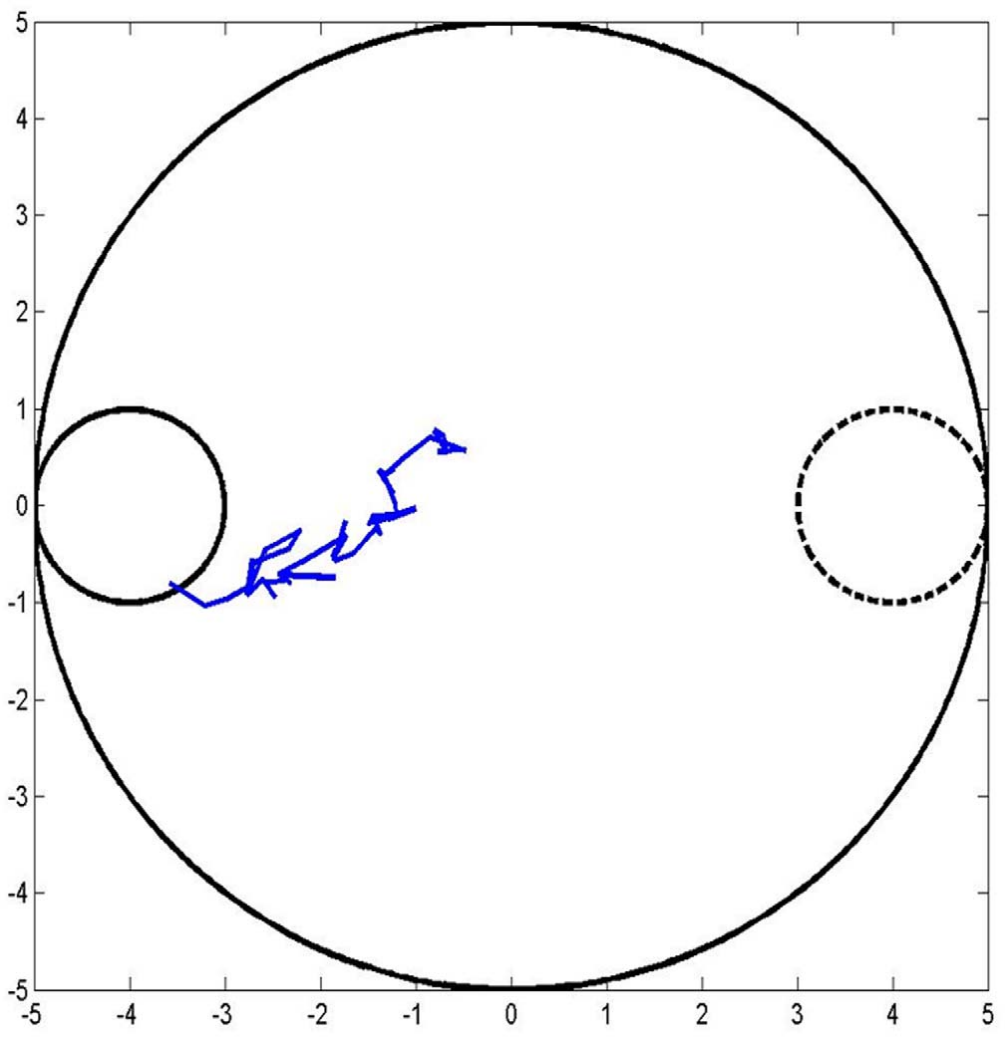
Sample trajectory of the simulated rat when only cue-based response assists navigation. Dotted circle refers to the previous location of the platform, while the solid circle on the left denotes the current location.

The second testing paradigm is equivalent to evaluating the performance of the agent when its hippocampus is lesioned in the experimental setup. In the model, the context-based response is completely suppressed, only cue-based navigation is used. Since the platform is now visible to the agent throughout the testing phase, it manages to reach the platform at the new location with considerable ease (fig. 10) compared to the previous case wherein cue-based response was not always selected. Average hit rate is 100% and the number of steps taken to reach the platform is 38, averaged over 50 trials. Under similar conditions of relocated platform, in experimental case, escape latency of the hippocampus-lesioned rat is lesser compared to a control rat [1], Similarly note that the model rat operating under purely cue-based strategy shows higher hit rate (100% after training) and fewer number of steps (44) to reach the platform, than the model that operates by competition between cue- and place-based strategies (87% hit rate, 129 steps).

### 3.2. Simulation of Parkinsonian Conditions

Parkinson's Disease (PD) is a degenerative disorder characterized by tremor, bradykinesia, postural instability and rigidity of muscles [42]. In PD, neurons in the SNc region of Basal ganglia degenerate, reducing the production and release of dopamine. PD-related motor impairment is thought to result due to disruption of dopamine signal from neurons of SNc [43].To simulate dopamine-deficient conditions, we constrain the upward fluctuations of δ, which signifies the phasic Dopamine signal, as follows:
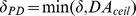
where min(x,a) is defined as:
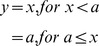
In the above equation, δ is the error signal directly calculated by eqn. (2.1), and δ_PD_ denotes a weakened dopamine signal. DA_ceil_ is chosen to be a value less than the maximum value of δ. Thus lesser values of DA_ceil_ denote a greater dopamine loss, and a smaller δ_PD_ than in normal conditions. A similar implementation of dopamine deficiency was used in The upper limit of 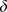, DA_ceil_, is gradually reduced and the performance of the rat in solving the navigation task is evaluated by determining escape latency for each value of 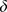. Dopamine for the cue-based module varies from −2.7 to 2.1 whereas for the place based module, it varies from −2.9 to 2.2. We rounded this off to a range of −3.5 to 3 to include the full range of variation in dopamine levels and then computed the DA_ceil_ commonly for both the modules as follows. The idea is that before PD conditions, DA_ceil_ is at the highest level, and there is zero cell loss; at the end of the duration of interest DA_ceil_ is at the lowest, and the cell loss is complete.
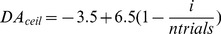
(3.1)The value of DA_ceil_ is mapped onto ‘%Dopamine cell loss’ as follows:
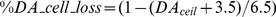
(3.2)


**Figure 11:**
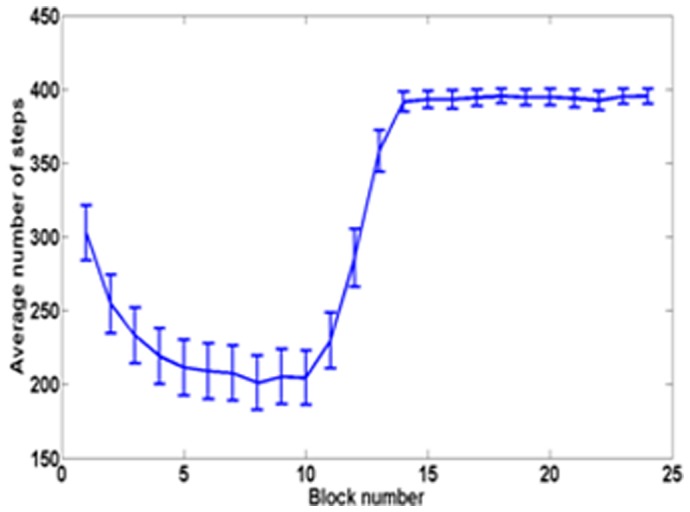
Average number of steps as a function of percentage Dopamine cell loss in PD model rat.

**Figure 12:**
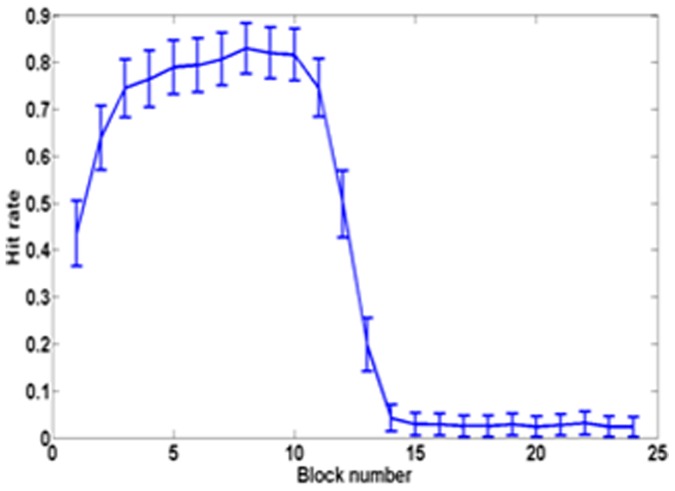
Hit rate as a function of percentage Dopamine cell loss in PD model rat.

**Figure 13:**
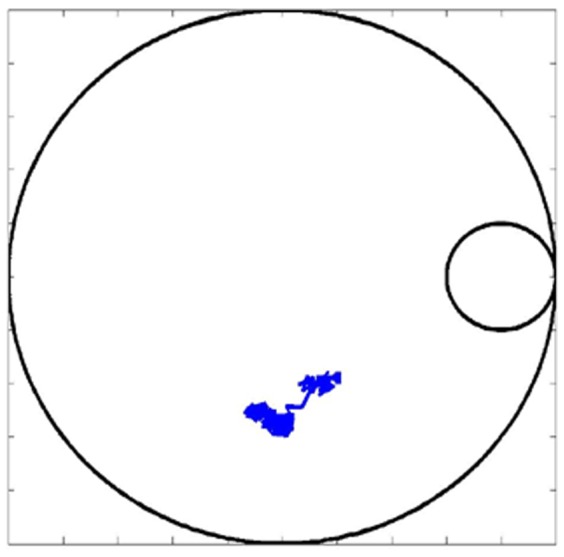
A sample trajectory of the PD model rat to reach the platform for % Dopamine loss = 50. The model rat's movements are confined to a small part of the pool, and show no consistent progression towards the platform.

The integrated system with random initial weights is trained for 100 trials while there is a progressive cell loss from 0% to 100%. The behavior of the simulated rat afflicted with PD is demonstrated for dopamine loss between 0 (intact basal ganglia) and 100 (total loss). It can be seen that the loss of dopamine does not affect learning or hit rate upto a certain level (around 40%). The average number of steps continues to decrease up to this point (fig. 11). Correspondingly, hit rate increases and remains constant till about 55% (fig. 12), beyond which there is a rapid deterioration of performance. Fig. 13 shows a sample trajectory of the model rat for cell loss of 50%.

## Discussion

The proposed model integrates hippocampus and basal ganglia into a single framework based on RL and explains experimental data in two instances. One of these experiments involves a navigation task in a Morris water maze, wherein the joint contributions of cue-based and place-based navigations are examined [1].The second experiment compares navigation performance of an MPTP rat with a control rat in a Morris water maze [44]. To our knowledge this is the first computational model of a PD/MPTP model rat navigating a water maze. Navigation models that incorporate hippocampus and basal ganglia do exist [5], [17], [18]. The novelty of the proposed model lies in that it is an extension of a novel line of research that proposes that the indirect pathway of basal ganglia serves a subcortical substrate for the Explorer component of RL [19]. In the specific context of navigation, it is suggested that the indirect pathway drives the exploratory activity necessary for navigation.

The model consists of separate modules for cue-based and place-based navigation systems. Each of these modules is an independent RL system, with its own critic, temporal difference (TD) error and mechanism for exploration. The neural substrates for these two modules are as follows. The critic for cue-based module is dorsolateral striatum with state information drawn from direct cortical inputs to this part of striatum. The critic for place-based module is dorsomedial striatum with the state information arising out of hippocampal projections to this part of striatum [1]. The TD error in both modules represents dopamine signals on nigrostriatal pathway, corresponding to specific projections to dorsomedial and dorsolateral parts of striatum. On the whole, the parallel basal ganglia loops that connect all basal ganglia nuclei, corresponding to dorsomedial and dorsolateral parts of striatum, may be thought of as two “copies of Basal ganglia,” subserving the two navigation systems.

The ultimate goal of our modeling efforts is not to develop an engineering or robotic system like, for example, [45], [46], that can navigate as efficiently as a biological system. Our goal is to develop a neurobiologically realistic model of basal ganglia, that can explain its role in navigation. But such a model seems quite ambitious in the context of the state of art of basal ganglia modeling. At the moment, even at systems level, with models involving abstract neurons, there are several “schools of thought” about basal ganglia function, without much coherence, with each group perfecting its own line of modeling. Even if we confine ourselves to Actor-Critic models of basal ganglia, there are so many variations, and diverse interpretations of Basal ganglia anatomy, that there is hardly any consistency [24].

When it comes to biophysical models of Basal ganglia, there exist a lot of models of single Basal ganglia nuclei, like, for example [47]. To our knowledge there is only one full-system, biophysical model of basal ganglia, that includes striatum, STN, GPe, GPi, dopamine signaling – all in a single model [48]. Even this model only captures certain firing patterns, and makes no attempt to link to any behavioral task. Therefore, to expect a detailed biophysical model of basal ganglia that captures neural firing patterns, and also explains behavioral results in navigation, in the current state of research, seems to be slightly unrealistic. In the current work, we present a network of simplified neuron models and link the network performance with behavioral results, though we reiterate that a biophysical model is the ultimate objective.

Another aspect of the present model that deserves to be taken up as a separate study in the future, is a quantitative comparison of the proposed model of exploration with similar models of exploration in navigation (like, eg, [5], and also basic mechanisms of exploration in RL (like, e.g., ε-greedy or softmax policy [49].Recently we applied the proposed basal ganglia model [21], to the card choice experiment used to study exploratory behavior by [50]. Performance of the proposed model closely resembles that of a RL-based behavioral model reported in [51].

In the proposed model, place-based strategy makes use of the spatial context encoded by the pattern of appearance of the poles that surround the pool. This approach to modeling place-based strategy is what [18], dubs as “place-recognition triggered response” and has been used in the past by several models of Hippocampus in spatial navigation [17], [45], [52], [53]. However, it must be noted that place-based navigation, ideally, would combine visual information with path integration (PI) information, and would be incorporated in future versions of our model.

Several models of Hippocampus assume that the location of the animal is known explicitly as (x,y) coordinates in some arbitrary, absolute coordinate system [17], [52]. But access to such information is artificial and unrealistic. In the present model, we extract spatial information from views which are based either on the visible cue, or the spatial context determined by the poles that circumscribe the pool. Such view-sensitive cells are indeed found in real hippocampal cells [54].

The present model has resemblances in its broad outlines to the spatial navigation model proposed by [17], which encompasses both cue-based and place-based strategies. However, the model of [17] does not clearly specify the mechanism of exploration, which is a key underlying mechanism of any form of navigation. Our group has been developing a line of basal ganglia models, that embody the hypothesis that the Direct Pathway part of basal ganglia subserves exploitation while the Indirect Pathway subserves exploratory behavior [19]. Successful navigation depends on the right mixture of goal-oriented and wandering/searching movements. Therefore, in the present model, as it was done earlier in a simpler model [23], we link the exploratory dynamics of Indirect Pathway with the wandering movements necessary for navigation. When an agent is introduced to a new environment, it first explores the environment and at later stages tends to exploit its knowledge about the environment, which is encoded in the internal representations of space.

Interestingly, the proposed approach to Basal ganglia modeling wherein the Direct Pathway subserves exploitation while the Indirect Pathway subserves exploration, has been applied in the past to explain a range of PD motor deficits like those observed in handwriting [55], reaching movements [22], and saccadic movements [56]. The same approach adapted to model avian homolog of basal ganglia, was able to explain impairments in bird song generation [57]. Therefore the uniqueness of the proposed model of the role of basal ganglia in navigation is that it is not a model that is exclusively developed for navigation; it is a general model of Basal ganglia that is shown to be consistently applicable for navigation also.

### Phasic and Tonic Dopamine

The proposed model has explicit representations for phasic and tonic dopamine signals, with the latter being an integrated version of the former (see eqn. (2.2)). Phasic and tonic Dopamine signals control the switch between Direct Pathway and Indirect Pathway in distinctive ways (see eqns. (2.3–2.4)).In experimental literature, phasic Dopamine release is thought to act on a time-scale of seconds, while tonic release acts over a few minutes [36]. Phasic release is linked to the difference in expected future reward and actual reward. Tonic and phasic dopamine releases are thought to have differential roles in updating of working memory information in the prefrontal cortex. Tonic Dopamine is thought to increase stability of maintained information in PFC by increasing the signal-to-noise ratio of the pattern to be stored. By contrast, phasic Dopamine is thought to determine *when* an activity has to be maintained or updated [58], [59]. It has also been suggested that tonic dopamine can regulate the intensity of phasic dopamine by the effect of the former on extracellular dopamine levels [60].

Niv et al [60], present a theory of phasic/tonic dopamine by invoking the notion of vigor of responding. Traditional RL-based theoretical models of dopamine emphasize the role of dopamine in learning. However, an aspect of behavior viz., vigor of response, which is observed to be affected by dopamine manipulations, is ignored by traditional models. Niv et al [61]proposed that tonic dopamine signal represents the average reward rate, 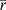, and were able to expand RL framework to explain not just action choice but also vigor of action. In the proposed model too, we have phasic dopamine denoted by δ, used for learning and switching between exploitation and exploration, and also tonic dopamine, 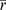, computed by summing past values of δ with a discount factor, and used to implement satiety. At high values of 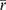, the simulated animal would have reached the goal and satiated; it therefore does not make any significant exploratory or exploitative movements.

### Navigation under Parkinsonian conditions

Parkinson's disease condition, which arises due to disruption of dopamine signal from the mesencephalic brain regions, has been simulated to compare the behavior of a normal agent adopting cue-based strategy of navigation and a PD-affected agent implementing the same. Results from simulation show that the capacity to accomplish the navigation task does not reduce till some critical level of dopamine loss reached beyond which it diminishes rapidly (fig. 11,12). These results are similar to those generally reported in the PD literature where the appearance of the symptoms of PD occurs only after 50–80% loss of substantianigra pars compacta cells [62]. Evaluation of cue-based performance of a simulated rat in a Morris water maze shows higher escape latency in the PD rat compared to a normal rat [44].

Section 2.3 presents an integrated model of navigation that incorporates both basal ganglia and hippocampus. A scheme for selecting between the actions suggested by the basal ganglia and hippocampus modules is described. The integrated system consists of two critics for the two forms of navigation considered. Echoing the conclusions of [1], we suggest that the value corresponding to the cue based or Stimulus-Response (S-R) type navigation is computed in the dorsolateral striatum and the value corresponding to place-based navigation is computed in dorsomedial striatum. (However, alternative substrates for these computations can also be suggested, as we discuss in the later part of this section). The basal ganglia and hippocampus modules are trained on specific days as in [1]. The integrated system captures the trends seen in the experiment of [1], which reveals the competition between the cue- and place-based navigation.

In the models of Sections 2, hippocampus is modeled as a combination of a SOM and a CANN. It may be argued that the transformation from view vector to CANN output is superfluous, and that the Value associated with hippocampus can be directly calculated from the Context-based Visual Input Vector representation. But there is evidence that suggests that hippocampus contains mechanisms for path integration [9]. Thus, incremental displacement (Δx, Δy) information must be integrated with the spatial information extracted from views, in order to construct a more reliable representation of space. CANN-based approaches have been suggested in the literature for this purpose [63]. Since we expect to incorporate path integration in our hippocampus model in the future, we used a combination of SOM and CANN to represent hippocampus in the present model.

### Towards a comprehensive understanding of the roles of Basal ganglia and Hippocampus in navigation

The precise form of cooperation between basal ganglia and hippocampus as described by [17], [52] and even the present work, wherein the hippocampus merely constructs a representation of space and offers it to basal ganglia for value computation, perhaps does not capture the complete story. Although the functional heterogeneities of striatum (dorsomedial striatum for spatial learning and dorsolateral for S-R type learning), as they are described in the present model, have significant experimental support, there are several exceptions to the rule. For example, in radial maze learning studies involving lesions of dorsal lateral and medial striatum [64] and lesions specific to dorsomedial striatum [65], hippocampus-dependent spatial learning was not impaired. Also in several other water maze studies [1],[66], dorsomedial striatal lesions did not completely block spatial learning.

The above studies reveal two things: 1) the picture of functional heterogeneity of striatum (dorsomedial striatum for spatial learning and dorsolateral for S-R type learning) is too simplistic. 2) hippocampus does not need to depend on the striatum for value computation, and for expression of spatial learning. These inferences urge us to search for alternative, broader perspectives of the nature of cooperation between basal ganglia and hippocampus in driving navigation.

If hippocampus does not need to depend on the striatum for value computation, then, is it possible that it can compute the value by itself? In other words, like the basal ganglia, does hippocampus contain complete reward processing machinery? The entorhinal cortex, considered the gateway to Hippocampus, receives inputs from amygdala and orbito-frontal cortex, which could potentially carry reward-related information into hippocampus [67]. Rolls & Xiang (2005) [54] found neurons in hippocampus that respond, not just to place, like the place cells, but to the combination of reward and place. These cells responded more to places that are associated with greater reward. The question that remains is: what is the precise signal that carries the relevant reward information to hippocampus? In basal ganglia, it is generally thought that dopaminergic projections from mesencephalic brain regions to the striatum carry reward signals. Does Hippocampus have similar sources of reward information? There is evidence supporting presence of mesencephalic dopaminergic projections to rat hippocampus [68]. Hippocampal neurons were found to express mRNA for D1- and D2-like receptors for dopamine [69]. Dopamine modulates neurotransmission in CA1 [70], and CA3 [71], regions of hippocampus. Cognitive deficits in PD patients have been linked to impairment of hippocampal long-term potentiation, a link that has been demonstrated by the fact that L-Dopa, a dopamine precursor, has been able to ameliorate the observed cognitive deficits [72].

**Figure 14:**
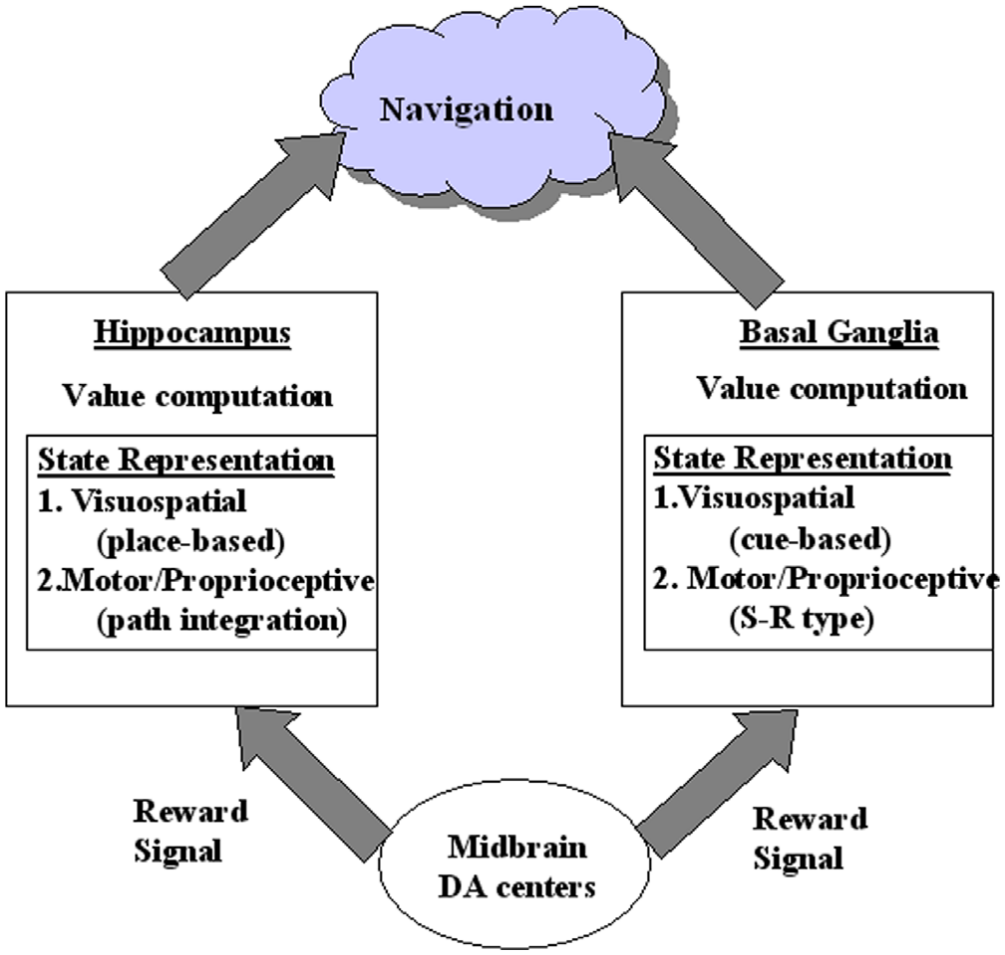
Schematic depicting a hypothetical, expanded view of the roles of basal ganglia and hippocampus to spatial navigation. In this view, both basal ganglia and hippocampus are capable of computing their own unique value functions by combining the respective sensory states accessible by them, and the dopamine projections from midbrain dopamine centers. Navigation subserved by basal ganglia based on visuospatial information is cue-based navigation. Navigation subserved by basal ganglia based on visuospatial information is S-R type navigation. Navigation subserved by hippocampus based on visuospatial information is place-based navigation. Navigation subserved by hippocampus based on proprioceptive information is path-integration.

The above experimental findings envisage a more expanded view of the contributions of basal ganglia and hippocampus in spatial navigation (fig. 14). The basal ganglia, as well as hippocampus, represents space in terms of both visuo-spatial and proprioceptive forms of sensory data. Basal ganglia uses its visuospatial representations for cue-based navigation. Likewise its proprioceptive and motor representations in striatum are perhaps used for S-R type or praxic form of navigation, which involves performing stereotyped body movements [73]. On the other hand, hippocampus uses its visuospatial information for constructing a spatial map of the surroundings, which is used for driving place-based navigation. The proprioceptive information received by hippocampus is used for path-integration. Thus, though basal ganglia and hippocampus have their unique mechanisms for representing space: basal ganglia's representation of space is probably closely tied to rewarding locations or cues, whereas the representation in hippocampus is based on a broader spatial context. Both basal ganglia and hippocampus probably construct their own internal Critics, using their own representations of the state and the reward-related information arising out of dopaminergic afferents. Competition between the navigational commands suggested by basal ganglia and hippocampus is perhaps settled by an appropriate form of gating (Fig. 14). Such an expanded view of the cooperation between basal ganglia and hippocampus might be able to explain the perplexing inconsistencies in experimental findings of spatial navigation [8].

## Supporting Information

Appendix S1 **List of parameter values used in the models described in various sections.** ‘r’ denotes ‘reward.’
(DOCX)
