# Supplementary material for: Modeling the Contributions of Basal Ganglia and Hippocampus to Spatial Navigation Using Reinforcement Learning
Source: PLoS One. 2012 Oct 26;7(10):e47467. doi: 10.1371/journal.pone.0047467 (PMC3482225; doi:10.1371/journal.pone.0047467)
Supplement: Appendix S1 — List of parameter values used in the models described in various sections. ‘r’ denotes ‘reward.’ (DOCX) [file pone.0047467.s001.docx]

Appendix:

List of parameter values used in the models described in various sections. ‘r’ denotes ‘reward.’

| parameter | Values used in place-based model | Values used in cue-based model | Values used in integrated model |
| --- | --- | --- | --- |
| γ | 0.95 | 0.95 | 0.95 |
|  | 1.7 | 1.6 | -- |
|  | 0.9 | 0.9 | 0.9 |
| β | -- | -- | 0.5 |
| η for training critic | 0.05 for r=1 | 0.01 for r=1 | 0.0005 for r = 2 (place-based module) |
|  | 0.0001 for r =-1 | 0.0001 for r=-1 | 0.00001 for r = -1 (place-based module) |
|  |  |  | 1e-6 for r = 2 (cue-based module) |
|  |  |  | 1e-7for r = -1 (cue-based module) |
